# Elliptical metallic rings-shaped fractal metamaterial absorber in the visible regime

**DOI:** 10.1038/s41598-020-71032-8

**Published:** 2020-08-20

**Authors:** R. M. H. Bilal, M. A. Saeed, P. K. Choudhury, M. A. Baqir, W. Kamal, M. M. Ali, A. A. Rahim

**Affiliations:** 1 Faculty of Electrical Engineering, Ghulam Ishaq Khan Institute of Engineering Sciences and Technology, Topi, 23640 Pakistan; 2 Division of Electronics and Electrical Engineering, Dongguk University, Seoul, 04620 Republic of Korea; 3 Institute of Microengineering and Nanoelectronics, Universiti Kebangsaan Malaysia, 43600 UKM Bangi, Selangor Malaysia; 4 Department of Electrical and Computer Engineering, COMSATS University Islamabad, Sahiwal, 57000 Pakistan; 5 Department of Engineering Sciences, University of Oxford, Park Road, Oxford, OX1 3PJ UK; 6 Department of Electronic and Computer Engineering, University of Limerick, Limerick, V94 T9PX Ireland

**Keywords:** Engineering, Optics and photonics

## Abstract

Achieving the broadband response of metamaterial absorbers has been quite challenging due to the inherent bandwidth limitations. Herein, the investigation was made of a unique kind of visible light metamaterial absorber comprising elliptical rings-shaped fractal metasurface using tungsten metal. It was found that the proposed absorber exhibits average absorption of over 90% in the visible wavelength span of 400–750 nm. The features of perfect absorption could be observed because of the localized surface plasmon resonance that causes impedance matching. Moreover, in the context of optoelectronic applications, the absorber yields absorbance up to ~ 70% even with the incidence obliquity in the range of 0°–60° for transverse electric polarization. The theory of multiple reflections was employed to further verify the performance of the absorber. The obtained theoretical results were found to be in close agreement with the simulation results. In order to optimize the results, the performance was analyzed in terms of the figure of merit and operating bandwidth. Significant amount of absorption in the entire visible span, wide-angle stability, and utilization of low-cost metal make the proposed absorber suitable in varieties of photonics applications, in particular photovoltaics, thermal emitters and sensors.

## Introduction

In recent years, optical metamaterials have gained considerable attention in both the engineering and scientific lexicons owing to the exotic electromagnetic (EM) response, that led to varieties of technological applications^1–6^. As has been in reports, these artificially engineered materials allow the versatile utility to manipulate the amplitude, phase, and polarization of the incidence radiation at a deep subwavelength scale^7^.

Metamaterials are generally comprised of nano-resonators, scatterers and meta-molecules of different size, shape, geometry, orientation, and arrangement. Within the context, the negative refractive index (RI)-based metasurfaces enable intriguing applications in super lensing^8^, planar filters^3^, optical cloaking^9,10^, wavefront manipulation^11,12^, optical chirality^13^, medical imaging^14^, and perfect absorption^15–17^. These are also tremendously exploited in various other EM applications, namely asymmetric transmission, plasmon-induced transparency, holography, and bio-sensing^6,17–19^.

Extensive studies have been reported on metamaterial absorbers operating in different frequency regimes^20–22^ owing to the prevalent applications in bolometer, holograms, stealth technology, solar energy harvesting, wireless communications, and sensors^15–17,23–27^. From the perspective of absorption bandwidth, the narrowband metamaterial absorbers covering the visible and infrared (IR) regimes find applications, such as thermal emission manipulation, nano-antennas, sensors, and resonators^28,29^. On the other hand, wideband absorbers have potentials in solar energy converters, artificial colors, thermal emitters, and many other optoelectronic applications^30,31^.

Within the context, photovoltaics have important roles in harvesting energy from the outdoor source (i.e., the sun) and artificial indoor light sources (namely, light-emitting diode, halogen, fluorescent and incandescent lamps) within the wavelength range of 300–3,000 nm^32^. Metamaterial-based absorbers have potentials in solar energy harvesting, and leave the possibilities of finding new alternatives for synthesizing relatively cheaper materials to achieve the purpose. Additionally, such metamaterials exhibit the application of plasmonic resonance in medical imaging^33^, photo thermal therapy^34^ and biosensing toward environmental protection^35,36^. In line with this, the two-dimensional (2D) materials have been greatly attracting as these open-up avenues for varieties of photonics-based applications – the feature basically due to strong absorption that happens because of their unique optical and electronic properties^37–40^.

To broaden the absorption spectrum, varieties of device configurations, including multilayer stacking of resonant elements in the vertical direction^41^, perpendicularly standing nanowires^42^ and multiple resonators using in-plane arrangement^43^, have been used. Within the context, Lai et al*.*^43^ used Al/SiO_2_/Al kind of sandwiched structures in a simple absorber with the integration of hybrid dual-resonators, and achieved polarization-insensitive absorption of above 95% in the 450–600 nm spectral range. Hoa et al*.*^41^ reported ultra-broadband metamaterial absorber with high absorptivity (> 90%) in the visible and near-IR regimes (480–1,480 nm) by adopting symmetrical metasurfaces in a periodic arrangement of multilayered conical frustums. A nearly similar type of 2D metamaterial absorber^31^ based on a dielectric-nickel grating structure was numerically investigated to demonstrate nearly *perfect* absorption at normal incidence in the visible regime (~ 400–800 nm). Zhang et al*.*^44^ achieved over 95% absorption of visible light by designing a dual-band metamaterial absorber comprising a five-layered metal-insulator-metal configuration. The absorber exploiting refractory materials in the metal-insulator composite stacks in ref.^45^ demonstrated an average absorption of ~ 97% under normal incidence.

All of the aforementioned techniques provide improved absorption bandwidth. However, complexities in structures introduce difficulties in manufacturing and applications. For example, the coalescing of multilayer absorbers with industrial technologies becomes challenging, owing to the bulky size, high manufacturing cost, and the use of noble metals^26,41–43^. In addition, though the use of multi-resonance approach remains simpler to fabricate the relevant device in comparison to the multilayer kind, the former method is still far behind the latter type owing to limited absorption in a specific frequency band^44,46^. Therefore, a relatively easy-to-fabricate absorber with enhanced absorption remains inevitable.

In this paper, we report a promising design of metamaterial absorber in the visible regime by integrating fractal periodic resonant structure to form the metasurface^47^. The self-similarity in fractal design can provide wideband absorption characteristics due to the multi-resonance phenomenon. To be more explicit, we provide the proof-of-principle demonstration of the absorber comprised of elliptical rings-shaped fractal metamaterial. We exploit tungsten (W) metal to form fractal designs of metasurface. This is because this metal is of low-cost, and also, shows high chemical stability that resists corrosion due to reactions with oxygen, acids and alkalis, thereby allowing usage in robust environment. Moreover, it has far higher melting point (~ 3,422 °C)^48,49^ than the other metals, namely gold, silver, copper, titanium and chromium – the feature that allows tungsten-based absorbers to endure elevated temperatures during high energy photon absorbance. The proposed wideband absorber shows average absorbance above 90% in the 400–750 nm wavelength span. Moreover, the absorption remains stable (above 70%) over a wide range of incidence obliquity (0°–60°) under the transverse electric (TE) mode. We also investigate the effects of operating conditions and geometrical parameters on the spectral response of the proposed absorber, which involves the analyses of performance characteristics evaluating the figure of merit (FOM) and operational bandwidth (OBW).

### Design and modeling

**Figure 1:**
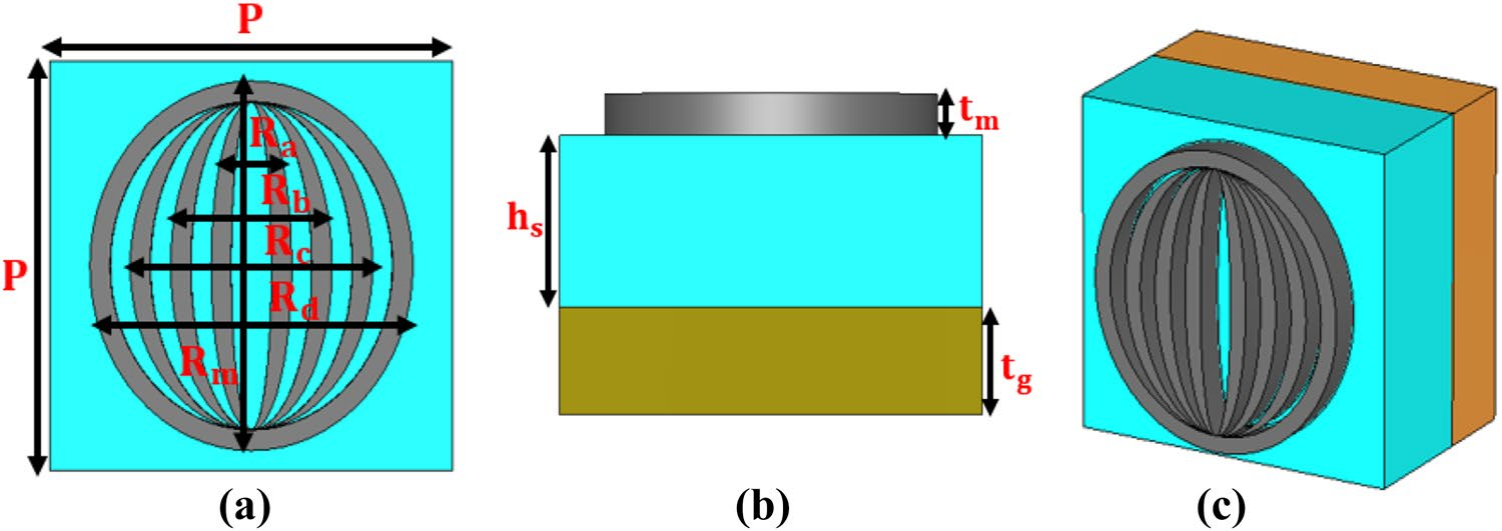
Schematic of the proposed FMA; (**a**) top-view (**b**) side-view, and (**c**) perspective view.

**Figure 2:**
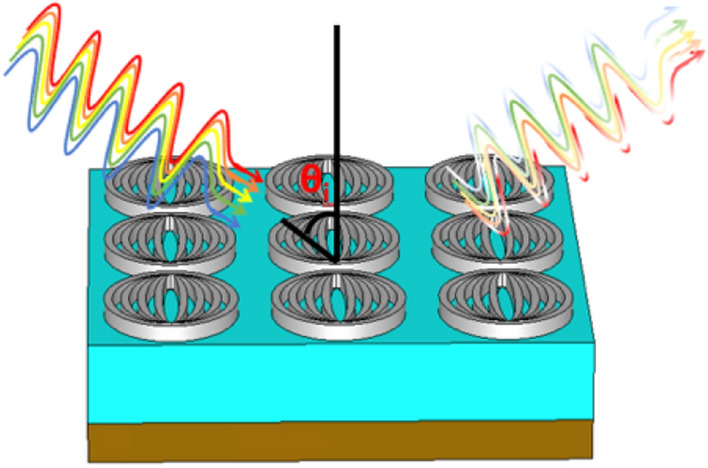
3D schematic of the proposed FMA.

Figure 1 presents the configuration of fractal metamaterial absorber (FMA); Figs. 1a–c, respectively, correspond to the top-view, side-view, and perspective view of the unit cell of the proposed FMA. Figure 2 exhibits the three-dimensional (3D) schematic of the FMA configuration. The top metasurface is comprised of elliptical rings-shaped meta-atoms of tungsten, separated by a silicon dioxide (SiO_2_) dielectric medium. At the bottom (below SiO_2_), we use a perfect electric conductor (PEC) to prevent transmission. As stated before, the reason for choosing tungsten is its excellent ability to endure high temperature, while absorbing high-energy photons. Likewise, SiO_2_ has a high melting point and low relative permittivity – the suitable properties that make this to be an effective dielectric layer.

The unit cell of metasurface consists of four elliptical resonators, connected internally with each other to form a fractal structure. Referring to Fig. 1, the optimized unit cell boundary is defined by $P = 200$ nm. Each tungsten elliptical ring is of 5 nm lateral thickness, and the major axis length $R_{m}$ of those four rings are 90 nm, 80 nm, 70 nm, and 60 nm with their respective minor axes lengths as $R_{d} = 80$ nm, $R_{c} = 60$ nm, $R_{b} = 40$ nm, and $R_{a} = 20$ nm (Fig. 1a). Also, $t_{m}$ and $h_{s}$ represent the thicknesses of the top metasurface and dielectric spacer, respectively. In our work, we vary the values of $h_{s}$ and $t_{m}$ in the ranges 50–80 nm and 15–30 nm, respectively. However, the respective optimized values of $t_{m}$ and $h_{s}$ are found to be 25 nm and 60 nm.

Within the context, fractals are self-similar repeated structures that usually contain two types of geometries, namely the deterministic fractal and random or statistical fractal. The deterministic fractals have specific dimension $D$ in fraction, calculated using the equation $D = \log n/\log M$ with *n* and *M* being the number of self-similar pieces and magnification factor, respectively. On the other hand, the random or statistical fractals (such as clouds, trees and coastline etc.) also have fractional dimensions, but there is no specific mathematical formula to calculate the dimensions^50^. In our work, the used elliptical rings-shaped structure in developing metamaterial belongs to the random fractal category, and we can only predict the dimension by visualizing the geometry. Obviously it has the fractal dimension in the range of $1 < D < 2$.

Under such geometrical parameters, we use the CST Microwave Studio to simulate the performance characteristics of the absorber. In this process, we employ the unit cell boundary conditions in the *x*- and *y*-directions, whereas the open add-space boundary conditions in the *z*-direction (the direction of wave propagation). When the incidence EM light falls upon the top metasurface from the + *z*-axis, it travels through the structure due to impedance matching (between the metasurface and free-space). The bottom groundsheet behaves as a perfect reflector to stop transmission (thereby making the parameter $S_{12} \approx 0$) and the middle dielectric substrate traps the light. The total absorbance $A\left( \lambda \right)$ can be written as $A\left( \lambda \right) = 1 - T\left( \lambda \right) - R\left( \lambda \right)$, where $T\left( \lambda \right)$ and $R\left( \lambda \right)$, respectively, represent the transmission and reflection, respectively. This equation can also be correlated with the $S$-parameters as $A = 1 - \left| {S_{11} } \right|^{2} - \left| {S_{12} } \right|^{2}$, where $S_{11}$ and $S_{12}$, respectively, correspond to the reflected and transmitted energies of the FMA. As stated before, the prefect reflector at the bottom layer completely blocks transmission, and therefore, the absorption can be evaluated from the $S_{11}$ parameter only.

## Results and discussion

The condition of impedance ($Z$) matching remains as the prerequisite for EM wave absorbers to exhibit the resonant behavior. Ideally, the device impedance, determined by $Z = \left( {\mu_{m} /\varepsilon_{m} } \right)^{1/2}$ ($\mu_{m}$ and $\varepsilon_{m}$ being the permeability and permittivity of medium, respectively), must match with that of the free-space (having the impedance $Z_{0}$ value as 377 Ω), in order to realize the perfect wideband absorbance with the minimal reflection in a specific operating wavelength span. The effective impedance $Z_{eff}$ can be calculated using the equation^21,22^

1 $$Z_{eff} = \sqrt {\frac{{(1 + { }S_{11} )^{2} - S_{12}^{2} }}{{(1 - { }S_{11} )^{2} - S_{12}^{2} }}} = \frac{{1 + {\text{S}}_{11} }}{{1 - {\text{S}}_{11} }}$$

**Figure 3:**
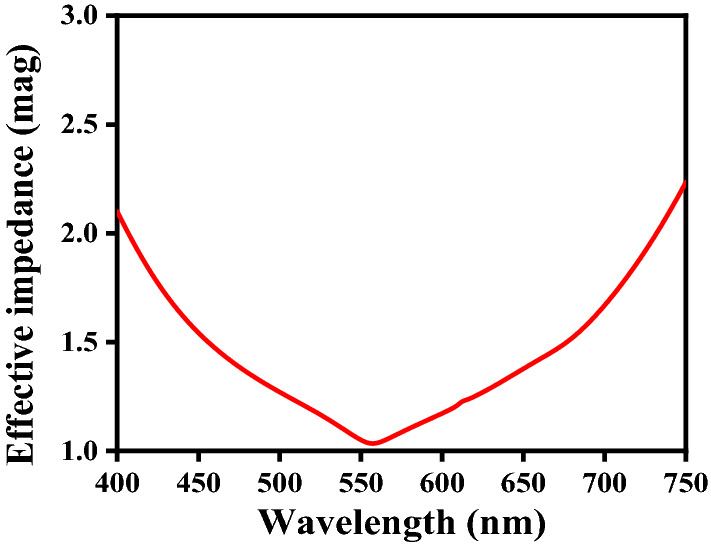
Wavelength-dependent effective impedance of the FMA structure.

Figure 3 shows the plot of effective impedance $Z_{eff}$ of the proposed FMA structure against wavelength $\lambda$. We observe that the wavelength-dependent effective impedance becomes nearly unity in ~ 560–570 nm range, i.e., the value of $Z_{eff}$ perfectly matches with that of the free-space in this wavelength span. Also, with the increase in wavelength, $Z_{eff}$ reduces from ~ 2.1 to ~ 1.0 until $\lambda = 560$ nm is reached. Upon further increasing the wavelength, $Z_{eff}$ shows an increase for $\lambda > 560$ nm, and $Z_{eff} \cong 2.2$ for $\lambda = 750$ nm. As such, the proposed FMA provides excellent impedance matching in a certain wavelength span in the visible regime.

**Figure 4:**
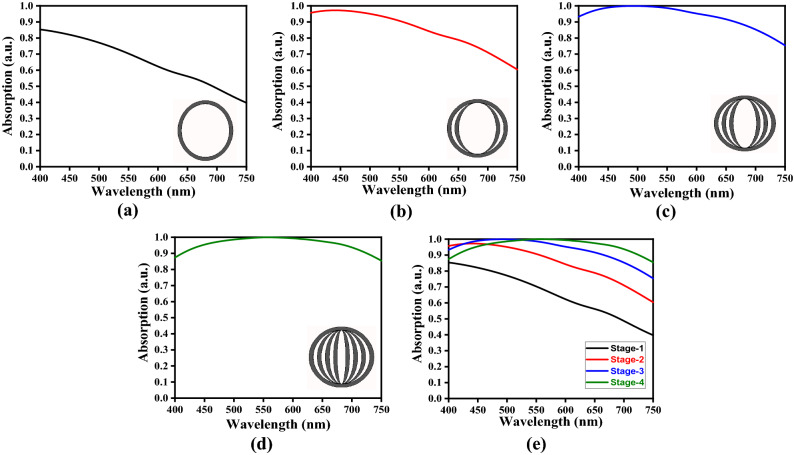
Absorption vs. wavelength plots corresponding to (**a**) Stage-1, (**b**) Stage-2, (**c**) Stage-3, and (**d**) Stage-4; (**e**) comparative look at the absorption spectra corresponding to all the defined stages.

We now investigate the absorption properties of the proposed FMA structure. We take the unit cell of metasurface as comprised of four internally connected elliptical resonators made of tungsten. However, we gradually increase the number of ring resonators in the metasurface assembly, and evaluate the wavelength-dependent absorption in every stage. We classify these stages as the Stage-1, Stage-2, Stage-3 and Stage-4, as the number of tungsten rings increases from 1 to 4 in the unit cell formation (in metasurface). Figure 4 illustrates the obtained results in respect of the absorbance–wavelength plots.

Considering the use of one elliptical ring (i.e., the Stage-1) as the resonator component in metasurface, Fig. 4a shows the wavelength-dependence of absorbance. We observe in this case the maximum absorbance to be ~ 85% at 400 nm wavelength, and the absorbance gradually decreases in a nearly linear form with the increase in wavelength, with its value being ~ 40% at 750 nm. When the resonator components assume two elliptical tungsten rings (i.e., the Stage-2) in the unit cell configuration, Fig. 4b exhibits the absorbance plots. We notice a significant increase of over 10% in absorbance in the entire wavelength span. Also, the absorption remains nearly 96% in the range of 400–450 nm (though it shows an increase of about 1% in this range), and then it gradually decreases in a nearly linear form to ~ 60% at 750 nm.

Significant increase in absorbance is further justified looking at the case of Stage-3, when the resonator components involve three tungsten elliptical rings in the assembly of resonator components in the unit cell. We notice the presence of perfect absorption (i.e., 100%) in the 465–530 nm wavelength range (Fig. 4c) in this case. Below this, the absorbance increases from ~ 94% (at 400 nm), and the increase in wavelength beyond 530 nm causes gradual decrease in it to ~ 75% at 750 nm. As such, we achieve the perfect absorption bandwidth of ~ 65 nm, which is fairly wide in nature. Upon further increasing the number of metallic elliptical rings (i.e., the Stage-4), the respective absorption spectrum in Fig. 4d exhibits perfect absorption in the range of 535–600 nm, i.e., with a bandwidth of ~ 65 nm. Before the lower limit, the absorption increases from ~ 89% (at 400 nm), and after the upper limit, the value becomes ~ 85% corresponding to 750 nm. As such, we find that the perfect absorption bandwidth is no more altered upon increasing the number of tungsten elliptical rings in the metasurface. However, the absorption band undergoes a red-shift of ~ 70 nm upon improvising the fractal metasurface from Stage-3 to Stage-4.

Figure 4e illustrates the absorption spectra for all the stages, as discussed above, in order to have a comparative look at the performance of the absorber. The enhancement in absorption with the elevating stages of fractal metasurface is clearly observed, which becomes more significant in the longer wavelength regime. As such, the introduction of elliptical rings strongly improvises the FMA structure to achieve broadband absorption characteristic due to the occurrence of uninterrupted plasmon resonances at the top fractal metasurface.

At this point, one would be interested in observing the effects on the absorption spectrum by using further higher stages of fractal designs in the metasurface, such as the Stage-5 or Stage-6. It must be mentioned at this point that the use of such higher stages does not exhibit good absorption, and therefore, we do not incorporate those results here. Moreover, implementing the Stage-5 and above will make the values of the major and minor axes of ellipses greater than that of the highest fractal stage in the current use (i.e., the Stage-4), thereby affecting the symmetry in the unit cell. In this work, our prime intention is to enhance the OBW of the absorber. To investigate this, we gradually increase the stages one-by-one, and determine the Stage-4 to be final one as it manifests good absorption as compared to the lower stages, such as the Stage-1, Stage-2 and Stage-3.

**Figure 5:**
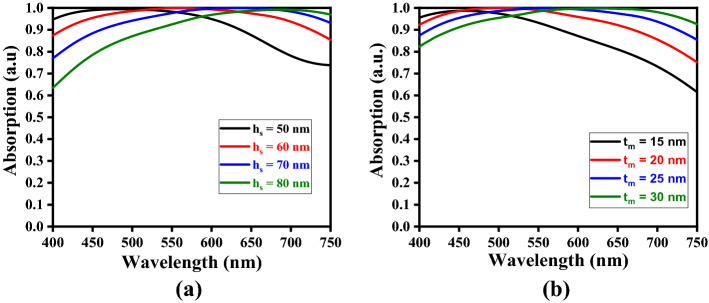
Wavelength-dependence of absorption corresponding to (**a**) different values of $h_{s}$ keeping $t_{m}$ fixed, and (**b**) different values of $t_{m}$ keeping $h_{s}$ fixed.

In the attempt of improving the wideband absorption characteristics, we now perform the study exploiting different parametric conditions of the FMA. In this stream, we obtain the results corresponding to different values of thicknesses $t_{m}$ (of the top metasurface) and $h_{s}$ (of the dielectric spacer). We first keep the value of $t_{m}$ fixed to 25 nm, and take $h_{s}$ as 50 nm, 60 nm, 70 nm, and 80 nm; Fig. 5a shows the obtained results. On the other hand, Fig. 5b corresponds to the absorption patterns obtained under varying values of $t_{m}$ (namely 15 nm, 20 nm, 25 nm, and 30 nm), while $h_{s}$ is kept fixed to 60 nm.

We observe in Fig. 5a that, corresponding to all the chosen values of $h_{s}$ (keeping $t_{m} = 25$ nm) the *perfect* absorption is achieved, the bandwidth of which remains almost unaltered (to a value ~ 50 nm) with the increase in $h_{s}$. However, the increase in $h_{s}$ results in significant amount of red-shift to the bandwidth position of perfect absorption. It is also noticeable from Fig. 5a that, corresponding to the highest value of $h_{s}$ (i.e., 80 nm), the absorption bandwidth is considerably reduced. This may be attributed to the extent of impedance matching and the trap of incidence radiation corresponding to higher dielectric thickness^51^. Also, the internal loss due to scattering related issues would influence the bandwidth.

On the other hand, the absorption spectra in Fig. 5b (with $h_{s}$ fixed to 60 nm) exhibit increase in the perfect absorption bandwidth with increasing $t_{m}$. The choice of $t_{m} = 15$ nm shows around 99% absorption in the visible wavelength band of ~ 450–470 nm (with a bandwidth of ~ 20 nm). The increase of $t_{m}$ to 20 nm yields perfect absorption in the wavelength span of ~ 470–540 nm with a bandwidth of ~ 70 nm. A further increase of $t_{m}$ to 25 nm results in 100% absorption in the ~ 520–595 nm band (i.e., the bandwidth becomes ~ 75 nm) in this case. Figure 5b also shows that $t_{m} = 30$ nm provides perfect absorption in the 575–670 nm, thereby giving the absorption bandwidth to be ~ 95 nm. It is noteworthy that, in describing such absorbers, the impedance matching remains the prime factor to obtain high absorption. We observe that the use of 25 nm thickness of metasurface yields fairly good matching of the metasurface impedance with that of the free-space (Fig. 3). That is the reason of obtaining excellent absorption in this wavelength span of 520–595 nm. Such observed perfect absorption bandwidths remain of very high value that can be useful for many photonics applications. The red-shift of absorption bands also happens with the increase in metasurface thickness – the feature attributed to the alteration of plasmon resonance.

At this point, it would be interesting to give a look at the other previously reported results on metamaterial absorbers so that a comparison can be made with the observations achieved in respect of the proposed FMA. Table 1 exhibits such a cursory description, taking into account the features of some of those and their relative merits and demerits. The last row in this table describes the work taken up in the present investigation.

**Table 1:** Comparison of the features of the previously reported metamaterial absorbers with the proposed FMA structure.

Metamaterial structure	Mediums used	Configuration	Dimension (nm^3^)	Absorption bandwidth (nm)	Remarks /limitation
Multiple stacked nanopillar arrays^52^	Si and Au	Multilayer	200 × 200 × 960	400–700	Costly fabrication
Multiple stacked square patches^53^	Al_2_O_3_ and Ag	Multilayer	250 × 250 × 320	460–600	Costly fabrication
Metallic gratings^54^	Ni	Monolayer	410 × 410 × 380	400–650	Easy fabrication
Fish-scale structure^55^	Quartz and Ag	Monolayer	380 × 380 × 235	400–667	Easy fabrication
Multiple layered hyperbolic metamaterials^56^	TiO_2_, Au, and BK7	Multilayer	Unknown	300–385	Costly fabrication, less bandwidth
Multiple stacked layers^57^	Si, Ag, and glass	Multilayer	Unknown	470–590	Costly fabrication, less bandwidth
Multiple hybrid dual resonators^43^	SiO_2_ and Al	Monolayer	400 × 400 × 130	435–615	Easy fabrication, less bandwidth
Modulated multistack grating^5^	SiO_2_, Au, Ag, Al and Ni	Multilayer	800 × 800 × 300	400–700	Large size, costly fabrication
Elliptical rings-shaped fractal structure [present work]	SiO_2_ and W	Monolayer	200 × 200 × 135	400–750	Small size, easy fabrication, large bandwidth

We attempted so far toward achieving wideband perfect absorption. However, the obtained results correspond to the situation of normal incidence (i.e., $\theta_{i} = 0^\circ$) of waves impinging on the fractal metasurface. In order to evaluate the prospective robustness of the proposed FMA, we now study the influence of incidence obliquity on the performance of the same. In the case of oblique incidence of waves, the angles of incidence and refraction essentially leave strong impact on the reflection coefficient, owing to the relation

2 $${\Gamma }_{ \bot } = \frac{{Z_{m} \cos \theta_{i} - Z_{0} \cos \theta_{t} }}{{Z_{m} \cos \theta_{i} + Z_{0} \cos \theta_{t} }}$$

where $\theta_{i}$, $\theta_{t}$ and $\theta_{r}$ are the angles of incidence, transmission, and refraction, respectively. Also, $Z_{0}$ and $Z_{m}$, respectively, represent the impedance values of the free-space and medium. According to Snell’s law,

3 $$\frac{{Z_{0} }}{{Z_{m} }} = \frac{{\sin \theta_{t} }}{{\sin \theta_{i} }}$$

Equations (2) and (3) provide the maximum absorption at the TE polarization as^58^

4 $$\left( {A_{{{\text{TE}}}} } \right)_{max} = \mu \varepsilon - \varepsilon^{2} \sin^{2} \theta_{i} - \mu^{2} \cos^{2} \theta_{i} = 0$$

**Figure 6:**
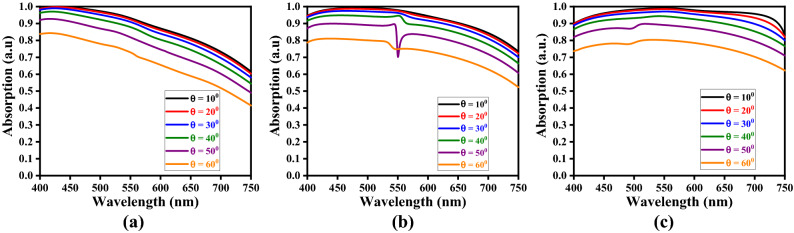
Absorption spectra for the values of $t_{m}$ as (**a**) 20 nm, (**b**) 25 nm, and (**c**) 30 nm, keeping $h_{s} = 50$ nm.

**Figure 7:**
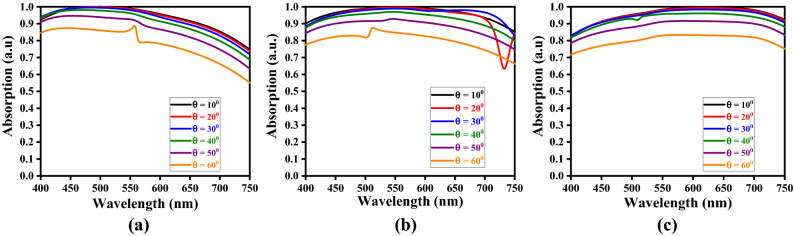
Absorption spectra for the values of $t_{m}$ as (**a**) 20 nm, (**b**) 25 nm, and (**c**) 30 nm, keeping $h_{s} = 60$ nm.

**Figure 8:**
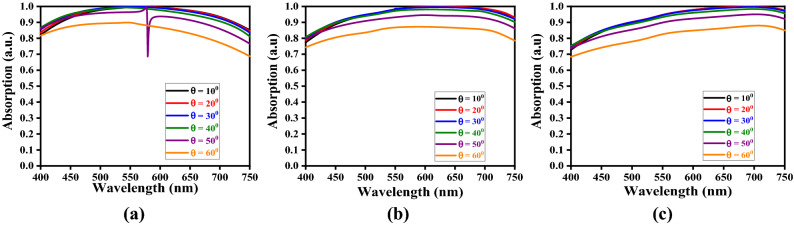
Absorption spectra for the values of $t_{m}$ as (**a**) 20 nm, (**b**) 25 nm, and (**c**) 30 nm, keeping $h_{s} = 70$ nm.

Now, for the computational purpose, we vary the incidence angle $\theta_{i}$ in the angular range of 10°–60° at a step of 10°, and observe the absorption spectra for different values of $h_{s}$ and $t_{m}$, keeping one of these parameters fixed. Also, we consider the Stage-4 kind of fractal design (of metasurface), as described before. For illustrative cases, we take three different values of $h_{s}$, viz. 50 nm, 60 nm, and 70 nm, and those of $t_{m}$ as 20 nm, 25 nm, and 30 nm; figs. 6, 7 and 8 exhibit the results in the form of wavelength-dependence of absorption spectra under different values of obliquity, and considering the TE-polarized incidence excitation.

Looking at figs. 6, 7 and 8, we find a kind of tread-off should be made in choosing the parametric values of $h_{s}$ and $t_{m}$, in order to attain wideband absorption characteristics. We observe the normal incidence of waves yields the maximum absorption. The anisotropy of structure and scattering of waves play important roles to reduce absorption with increasing obliquity. For low values of dielectric layer thickness, the absorption keeps on decreasing with increase in wavelength (Fig. 6a). The increase in metasurface thickness resolves this issue, as can be seen in figs. 6b and 6c, thereby yielding fairly stable wideband operation. However, band-notch appears with increase in metasurface thickness to 25 nm – the feature that is eliminated upon further increasing $t_{m}$. Such dips in absorption pattern indicate relatively weak coupling of incidence radiation with metasurface.

It also becomes obvious from figs. 6, 7 and 8 that, for a certain value of metasurface thickness, the increase in dielectric layer thickness $h_{s}$ results in enhanced absorption. However, once again, a suitable tread-off between $h_{s}$ and $t_{m}$ would yield the maximum absorption with increased wideband characteristics. Among the results depicted in these figures, the parametric conditions used in Fig. 7c and 8b present fairly well wideband nature of absorption spectra; in all the cases, however, the normal incidence excitation provides the maximum absorption. This is attributed to the fact that, under the TE-polarized excitation, the incidence radiation remains parallel to the elliptical rings-based fractals, thereby allowing enhanced absorbance of the proposed structure.

**Figure 9:**
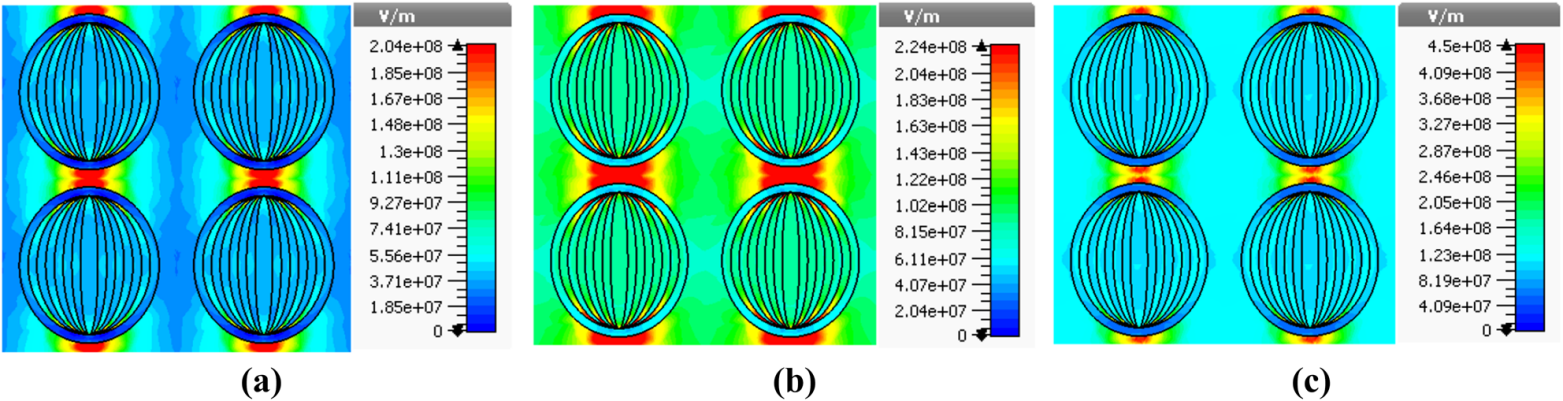
Electric field distribution patterns corresponding to absorptions at (**a**) 400 nm (**b**) 600 nm, and (**c**) 750 nm.

**Figure 10:**
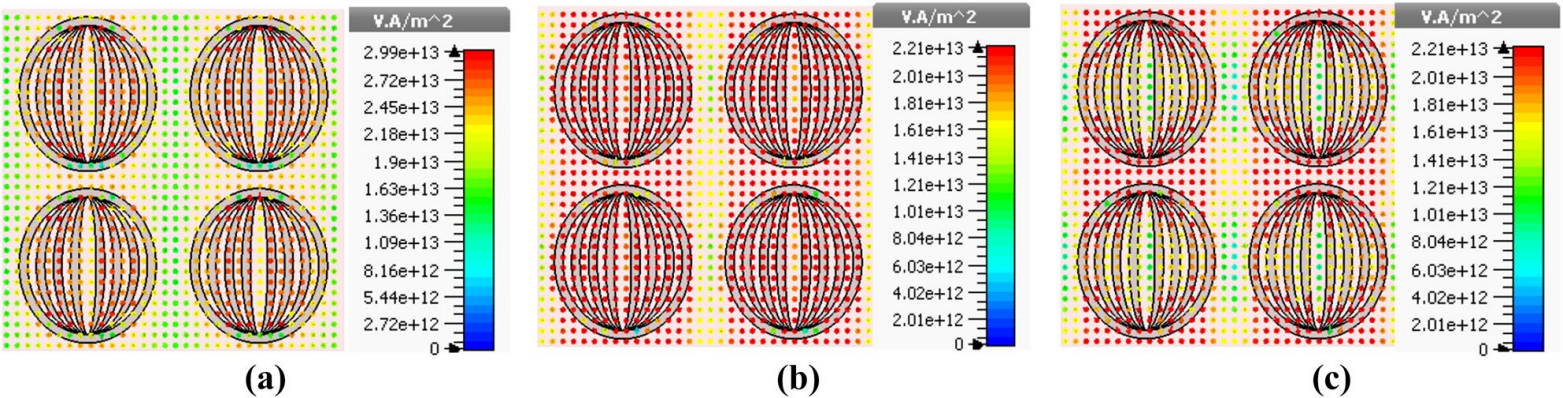
Surface power flow patterns corresponding to absorptions at (**a**) 400 nm (**b**) 600 nm, and (**c**) 750 nm.

With the aim of investigating the physical mechanism of absorption, we plot the electric field distribution patterns. For this, we consider the Stage-4 fractal design, and present such patterns under the normal incidence of waves for the wavelength values as 400 nm (Fig. 9a), 600 nm (Fig. 9b) and 750 nm (Fig. 9c). Also, these plots correspond to the parametric values $h_{s} = 60$ nm and $t_{m} = 25$ nm. With the use of such a geometry (of absorber) and the operating condition, Fig. 10 illustrates the surface power flow patterns corresponding to the stated values of incidence wavelengths. These figures show how the resonance conditions facilitate localizing the energy of incidence radiation.

It is evident from Fig. 9 that the electric field is primarily localized around the metallic fractal designs, particularly at the top and bottom edges of the same, thereby resulting in enhanced absorption. Among the three situations, we observe that the field strength remains maximum in Fig. 9b, that corresponds to 600 nm operating wavelength. This can be justified upon looking at the results in Fig. 5b as well, which exhibits the maximum absorption at 600 nm wavelength, when the absorber assumes the aforementioned parametric values. Within the context, the localization of electric field specifies the existence of resonance cavity modes due to the fractal nature of metasurface, as the incidence radiation is successfully trapped at the top of metal–dielectric interface^47,51^. The strong magnetic resonance takes place due to the localized surface plasmon resonance effects. The field distribution patterns indicate the elliptical rings-shaped structure in metasurface could support in confining and then effectively absorb the incidence light by the absorber.

**Figure 11:**
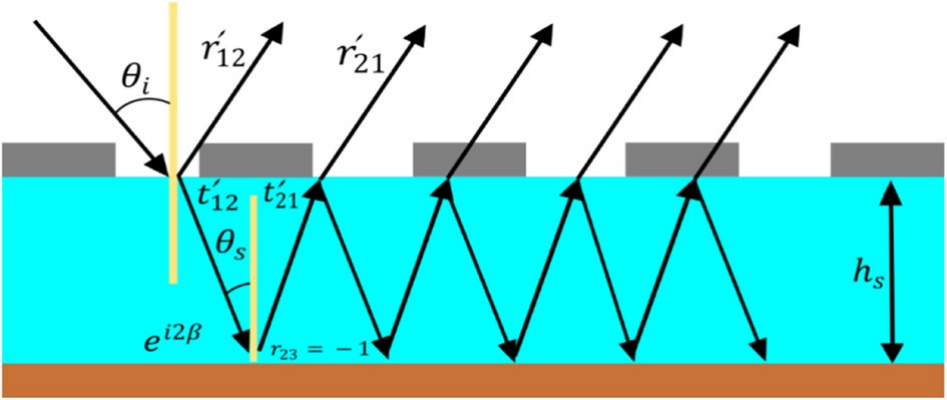
Fabry-Pérot cavity kind of model of the proposed absorber.

We now perform quantitative analysis to verify the absorption mechanism of the proposed FMA. For this purpose, we exploit the theory of interference (of light)^59,60^ considering the metasurface as a Fabry-Pérot kind of resonance cavity, as shown in Fig. 11. In this figure, the air-spacer interface is having an array of elliptical rings-based resonator components that function as the impedance-tuning surface. The ground plane serves as a perfect mirror with the reflection coefficient as $- 1$. Due to insignificant near-field interaction between the top metasurface and ground plane, however, only linked through the multiple internal reflections, we consider the Fabry-Pérot kind of model as a decoupled system.

In our computations, we take the top resonators and ground plane as zero thickness surfaces. As Fig. 11 exhibits, an incidence EM ray undergoes partial reflection (in the air) and transmission (into the spacer) upon interacting with the fractal metasurface. The respective reflection and transmission coefficients can be written as $r_{12}^{\prime } = r_{12} e^{{i\phi_{{{\text{r}}12}} }}$ and $t_{12}^{\prime } = t_{12} e^{{i\phi {\text{r}}_{12} }}$ (Fig. 11); $\phi$ being the phase of wave.

The transmission coefficient continues to alter with the complex propagation wavenumber $\beta = nk_{0} h_{s}$ ($k_{0}$ being the free-space wavenumber), meets the ground plane, and reflects back to the spacer with the reflection coefficient of $- 1$. Similarly, another form of partial reflection and transmission occurs with reflection and transmission coefficients given as $r_{21}^{\prime } = r_{21} e^{{i\phi_{{{\text{r}}21}} }}$ and $t_{21}^{\prime } = t_{21} e^{{i\phi {\text{r}}_{21} }}$, respectively. These multiple reflections result in a phase shift of $\beta$ that contributes to the destructive interference, thereby trapping the incidence radiations inside the absorber, and providing the maximum amount of absorption. The overall reflectance can be written as^60,61^:

5 $$r = r_{12}^{\prime } - \frac{{t_{12}^{\prime } t_{21}^{\prime } e^{i2\beta } }}{{1 + r_{21}^{\prime } e^{i2\beta } }}$$

**Figure 12:**
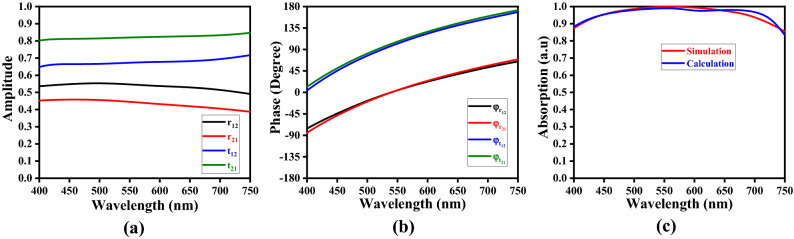
Plots of (**a**) amplitude, (**b**) phase, and (**c**) absorption (against wavelength) of the proposed FMA.

Using Eq. (5), the total absorption $A$ can be obtained from the equation $A = 1 - \left| r \right|^{2}$. Figure 12 depicts the wavelength-dependence of the magnitudes of amplitude (Fig. 12a), phase (Fig. 12b) and absorption (Fig. 12c) of the proposed absorber, as obtained through computations. Figure 12c shows the numerical results to be in strong agreement with those obtained through simulations, thereby justifying the correct use of the Fabry-Pérot kind of model in determining the absorption characteristics of the FMA.

In order to investigate the performance characteristics of the proposed FMA, we analyze the FOM and OBW of it. The results can be optimized by using the best suitable values of these two parameters related to the operating features. The FOM, shown by $\eta_{OBW}$, can be related to OBW ${\Delta }\lambda$ through the equations^62^

6 $$\eta_{OBW} = \frac{1}{{{\Delta }\lambda }}\mathop \smallint \limits_{{\lambda_{min} }}^{{\lambda_{max} }} A\left( {\lambda ,\theta_i } \right)d\lambda$$

where

7 $$\Delta \lambda = OBW = \lambda_{max} - \lambda_{min}$$

**Figure 13:**
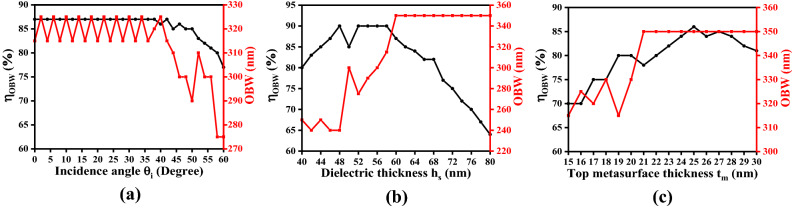
Plots of FOM and OBW against (**a**) incidence angle $\theta_{i}$, (**b**) dielectric spacer thickness $h_{s}$, and (**c**) top metasurface thickness $t_{m}$ of the proposed FMA.

In Eq. (6), $A\left( {\lambda ,\theta_i } \right)$ represents absorption that essentially depends on the incidence angle. Also, $\lambda_{min}$ is the minimum operating wavelength which is kept fixed, and the maximum value of wavelength $\lambda_{max}$ depends on the absorption threshold. In the present work, we keep $\lambda_{min}$ fixed to 400 nm, whereas the value of $\lambda_{max}$ depends on the threshold absorption condition. Figure 13 exhibits the plots of $\eta_{OBW}$ and OBW under different parametric and operating conditions of the FMA using the TE-polarized incidence excitation.

In order to optimize the incidence angle $\theta_{i}$, we plot $\eta_{OBW}$ and OBW as a function of $\theta_{i}$, keeping the other parametric values fixed; Fig. 13a shows the obtained results. In such computations, we take $h_{s} = 60$ nm, $R_{m} = 90$ nm, $R_{a} = 20$ nm, $R_{b} = 40$ nm, $R_{c} = 60$ nm, $R_{d} = 80$ nm, and $t_{m} = 25$ nm. We observe in this figure that the best possible value of FOM remains ~ 87.5% corresponding to $\theta_{i}$ in the 0°–35° range, which yields the OBW as ~ 325 nm. Upon increasing $\theta_{i}$, the value of OBW shows gradual decrease, and becomes ~ 305 nm for 60° incidence obliquity.

We next attempt to optimize the dielectric spacer thickness $h_{s}$, keeping the other parameters and operating conditions fixed; Fig. 13b exhibits the plots of $\eta_{OBW}$ and OBW under normal incidence (i.e., $\theta_{i} = 0^\circ$) in the range of $h_{s}$ as 40–80 nm. We observe in this figure the highest value of $\eta_{OBW}$ to be ~ 90% corresponding to $h_{s} = 48$ nm, and also, in the span ranging from 52–58 nm. The highest value of OBW is about 450 nm, which can be achieved for $h_{s}$ in the range of 60–80 nm. However, considering the highest value of $\eta_{OBW}$, the choice of $h_{s}$ above 60 nm should yield excellent performance of the proposed FMA under the condition of normal incidence.

Figure 13c illustrates the plots of $\eta_{OBW}$ and OBW under varying fractal metasurface thickness $t_{m}$ in the range of 15–30 nm. This figure shows the results related to the optimization of $t_{m}$, keeping the other parametric values and operational conditions fixed. These results correspond to the normal incidence of waves. It becomes obvious from this figure that the FOM attains the maximum value as 86%, which is achieved for $t_{m} = 25$ nm. Also, the value of OBW remains 350 nm for a range of $t_{m}$ from 21 to 30 nm. Figure 13 shows that the proposed FMA yields an OBW of 350 nm corresponding to the metasurface thickness 21–30 nm and dielectric spacer thickness 60–80 nm under the condition of normal incidence excitation. Using this performance analysis, the absorption characteristics of the proposed FMA can be improved by carefully choosing the optimized geometrical parameters of the unit cell.

## Conclusion

From the above discussions, it can be inferred that the use of arrayed tungsten elliptical rings-based fractal metasurafce in absorber configuration would yield wideband spectral absorption characteristics in the visible regime. The results indicate over 90% absorption in the wavelength span of 400 ≤ λ ≤ 750 nm. To be more explicit, upon increasing the metasurafce thickness from 15 to 30 nm, over 99% absorption can be achieved with the respective values of absorption bandwidth increasing from ~ 20 nm to ~ 95 nm. In particular, the choice of 25 nm metasurface thickness results in *perfect* absorption in the ~ 520–595 nm band with a fairly large bandwidth of ~ 75 nm – the feature that can be useful for many photonics applications. Also, the increase in metasurface thickness causes alteration of plasmon resonance condition, thereby resulting in red-shift of absorption bands. The incidence obliquity affects the magnitude of absorption retaining the wideband characteristic. The case of normal incidence, however, exhibits maximum absorption; the increase in incidence angle from 10° to 60° drops absorption by ~ 20%. The use of analytical approach exploiting the Fabry-Pérot theory of interference phenomenon gives results in strong agreement with those obtained through simulations, thereby justifying the use of Fabry-Pérot model in treating the proposed FMA structure. The study also incorporates optimization of geometrical parameters and operating conditions of FMA through determining the FOM and OBW. The results reveal improved absorption characteristics with an OBW of 350 nm corresponding to the metasurface thickness in the range of 21–30 nm and the dielectric layer thickness in the range of 60–80 nm under the normal incidence of waves.
